# Supplementary material for: SARS-CoV-2 Infection of Lung Epithelia Leads to an Increase in the Cleavage and Translocation of RNase-III Drosha; Loss of Drosha Is Associated with a Decrease in Viral Replication
Source: Genes (Basel). 2025 Oct 20;16(10):1239. doi: 10.3390/genes16101239 (PMC12564117; doi:10.3390/genes16101239)
Supplement: Supplementary file 1 [file genes-16-01239-s001.zip › genes-3924372-supplementary.pdf]

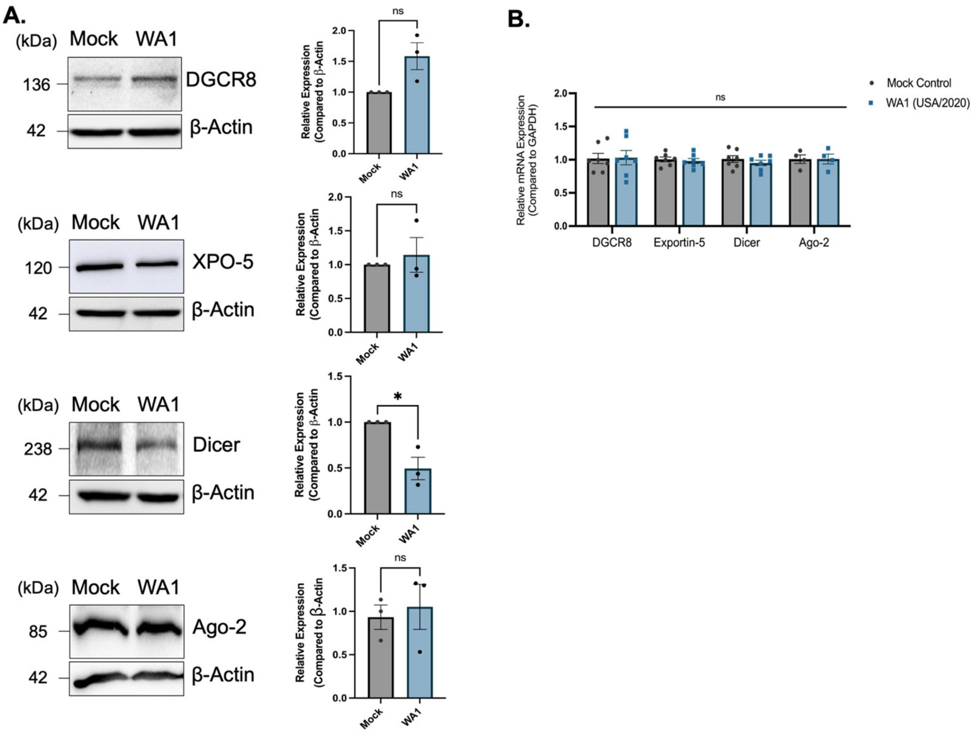

Supplementary Figure S1. The expression of key proteins within the miRNA biogenesis pathway, at the mRNA and protein level, following SARS-CoV-2 infection. (A) Western Blot of four proteins within the miRNA biogenesis pathway in mock and SARS-CoV-2-infected (MOI 1.0) NuLi-1 cells. Dicer expression is significantly decreased following infection. (B) qRT-PCR of the mRNA transcripts of four key proteins within the miRNA biogenesis pathway. Following infection there is no significant change in expression at the transcript level. Data are normalized to Beta-Actin or GAPDH and are presented as mean fold change ± SD from at least three independent experiments. The statistical significance between groups was determined using a two-tailed students T test, or two-way ANOVA followed by Tukey's post hoc test (ns, not significant; $*p < 0.05$).

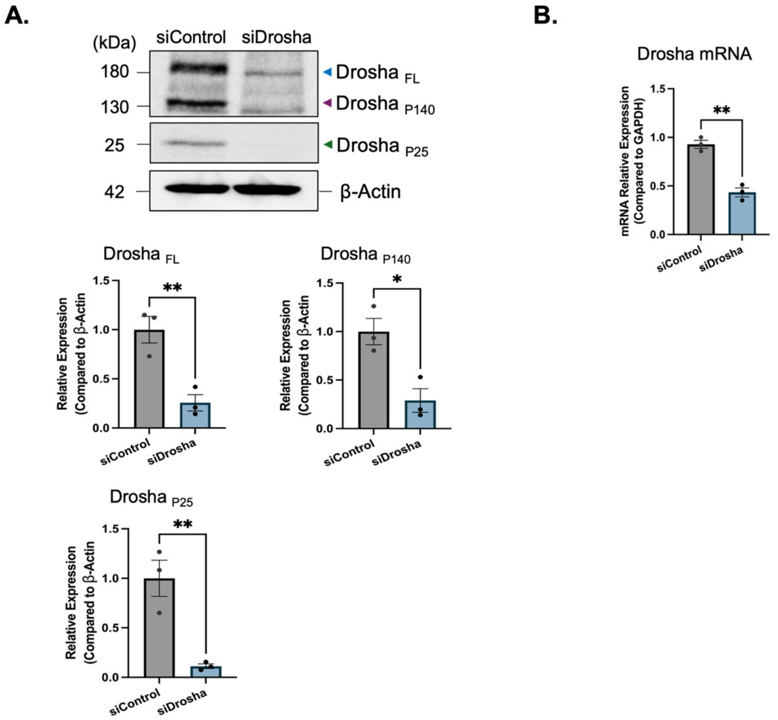

Supplementary Figure S2. siRNA knockdown confirmation of Drosha p140 and p25 isoforms. (A) Western blot validation of Drosha isoforms by siRNA knockdown. NuLi-1 cells were transfected with 100pmol Drosha-specific siRNA (siDrosha) or a non-targeting control siRNA (siControl). Western blotting shows a marked reduction in both FL and cleavage product (P140 and P25) bands in siDrosha-treated cells, confirming their identity as Drosha-specific. (B) qRT-PCR quantification showed a significant reduction in Drosha mRNA expression. Data are normalized to Beta-Actin or GAPDH and are presented as mean fold change ± SD from at least three independent experiments. The statistical significance between groups was determined using a two-tailed students T test (*$p < 0.05$; **$p < 0.01$).

Supplementary Table S1. Cell lines used in this study.

Cell Line	Cell Type	Sex	Modifications
NuLi-1	Bronchial; *Homo sapiens*	Male	Normal; hTERT
VERO E6-hACE-2-hTMPRSS2	Kidney; *Ceropithecus aethiops*	-	hACE-2/hTMPRSS-2
MRC-5	Lung fibroblast; *Homo sapiens*	Male	Normal; primary
HCT-116 (WT)	Colorectal Carcinoma; *Homo sapiens*	Male	Ras codon-13 mutation;
HCT-116 (Drosha KO)	Colorectal Carcinoma; *Homo sapiens*	Male	Mutated Ras; Drosha CRISPR

Supplementary Table S2. Antibodies used in this study.

Name of Antibody	Manufacturer; Catalogue #	Species; Clonality	Dilution; Application
Drosha Antibody #1	CST; 3364S	Rabbit; Monoclonal	1:1000 (WB) 1:50 (ICC)
Drosha Antibody #2	Santa Cruz; SC-393591	Mouse; Monoclonal	1:1000 (WB)
Drosha Antibody #3	Abcam; AB12286	Rabbit Monoclonal	1:1000 (WB) 1:50 (ICC)
Argonaute-2	Sigma; SAB4200085	Rat; Monoclonal	1:1000 (WB)
DGCR8	CST; D78E4	Rabbit; Monoclonal	1:1000 (WB)
Dicer	Santa Cruz; SC-136979	Mouse Monoclonal	1:1000 (WB)
Exportin-5	Santa Cruz; SC-271036	Mouse Monoclonal	1:1000 (WB)
Beta Actin	Santa Cruz; SC-47778	Mouse Monoclonal	1:1000 (WB)
HRP-Linked Anti-Rabbit	CST; 5127S	Mouse Monoclonal	1:1000 (WB)
HRP-Linked Anti-Mouse	CST; 58802	Rabbit; Monoclonal	1:1000 (WB)
HRP-Linked Anti-Rat	CST; 7077S	Goat; Monoclonal	1:1000 (WB)
AlexaFluor 488	CST; 4412S	Goat; Monoclonal	1:1000 (ICC)

Supplementary Table S3. q/RT-PCR primer sets used in this study.

Primer Name	Sequence (5′ – 3′)
GAPDH FWD	CCACTCCTCCACCTTTGAC
GAPDH REV	ACCCTGTTGCTGTAGCCA
SARS-CoV-2 ORF1a FWD	TTCAGTTGACTTCGCAGTGG
SARS-CoV-2 ORF1a REV	GGACGGGTTTGAGTTTTTCA
SARS-CoV-2 N Gene FWD	ATGCTGCAATCGTGCTACAA
SARS-CoV-2 N Gene REV	GACTGCCGCCTCTGCTC
SARS-CoV-2 5′UTR FWD	ACTGTCGTTGACAGGACACG
SARS-CoV-2 5′UTR REV	AACACGGACGAAACCGTAAG
HCoV-229E N Gene FWD	TCAATCTCGGAATCCTTCAAGT
HCoV-229E N Gene REV	AGGCTTAGGAGTACCCGTTTTC
HCoV-229E 5′UTR FWD	CTTTGATGCTGGAGTCGTAGTG
HCoV-229E 5′UTR REV	GGCGTTTGTAGAATGGAATCTC
HCoV-OC43 N Gene FWD	AGGCTATTCCGACTAGGTTTCC
HCoV-OC43 N Gene REV	TACTACGCGATCCTGCACTAGA
SINV N Gene FWD	GGTTCCTACCACAGCGACGAT
SINV N Gene REV	TGATACTGGTGCTCGGAAAACA
IFN-β FWD	AAGCTCCTGTGGCAATTGAATG
IFN-β REV	TCTCATAGATGGTCAATGCGGC
CXCL10 FWD	GCTCTACTGAGGTGCTATGTTC
CXCL10 REV	GGAGGATGGCAGTGGAAGTC
Drosha AS27a FWD	TGTGCATCTTGATTTGCATCTG
Drosha AS27a REV	GGAGAAGTTTCAATAAGTTGTCG
Drosha AS32a FWD	TGGTGTTGAAAATGGCAAGAC
Drosha AS32a REV	TGGAAAGAAGCAGACATTCAT
Drosha FL (ORF) FWD	TCACGCCTTGGCCAAGATGAC
Drosha FL (ORF) REV	GCTGATTCTGAACAATGGCAG
Drosha (Exons 1-3) FWD	GAAGAGTTTTCCCTCCCTTGG
Drosha (Exons 1-3) REV	CACATCCCCGGGAAAAGC
Drosha (Exons 5-8) FWD	CGACAGGAGAAGACAAGACAGC
Drosha (Exons 5-8) REV	GTGCATCCAGCAGGTTCAGG
Drosha (Exons 31-35) FWD	CAGCGCTGTACATTGATAAGGA
Drosha (Exons 31-35) REV	ATCTGGCTCTCTCTCTTGATGC
Drosha Tile 1 (199-1447nt) FWD	GGGGATGTGAAGGATACAGAAA
Drosha Tile 1 (199-1447nt) REV	GCTCTCTTCTTCTCCCTACTTGG
Drosha Tile 2 (1137-2524nt) FWD	AGACAAGACAGCCGGTACAGAT
Drosha Tile 2 (1137-2524nt) REV	ATATTGGCAATCTCCTCCTCAG
Drosha Tile 3 (2161-3615nt) FWD	TTGAAGGATTTTCTATGTTTGCAC
Drosha Tile 3 (2161-3615nt) REV	GTGGGTGGAGAGGATAATTGAG
Drosha Tile 4 (2912-4460nt) FWD	CATCCGTTCTGATGTCTGTCA
Drosha Tile 4 (2912-4460nt) REV	ATCTGGCTCTCTCTCTTGATGC
DGCR8 FWD	GCCTCCTCATAGACCCGAAC
DGCR8 REV	CCACTGACAGGGCTAAGGAG
Exportin-5 FWD	CAACTCCACCCAGCGCTAC
Exportin-5 REV	TGACAACGTGTTCCAGGATCT
Argonaute-2 FWD	TTTTGAGACGATCCAGGCCC
Argonaute-2 REV	TTTTCCAGAGAGAAGGCCGG
Dicer FWD	TACCCGTGCAACCAACTACC
Dicer REV	GCATTACGGCCATCACAGGA

Supplementary Table S4. Sequences of siRNAs used in this study.

siRNA	Targeted Region	Strand	Strand	Sequence (3′ – 5′)
siDrosha	3′UTR	Guide	Sense	C.A.U.U.A.G.A.A.U.A.G.G.A.G.A.G.A.A.A.U.U
		Passenger	Antisense	U.U.U.C.U.C.U.C.C.U.A.U.U.C.U.A.A.U.G.U.U
